# Supplementary material for: Development of a computer-aided design software for the quantitative evaluation of aesthetic damage
Source: PLoS One. 2019 Dec 18;14(12):e0226322. doi: 10.1371/journal.pone.0226322 (PMC6919621; doi:10.1371/journal.pone.0226322)
Supplement: S1 Code site — 1. (ZIP) [file pone.0226322.s001.zip › [EESC_jr][Projeto]Relatorio_07_21/reports/templates_zip/backyard-high-converting-landing-page-template.zip!/backyard/backyard/app.html]

Backyard App Landing Page


# Mobile Strategy App

Backyard is a modern and customizable landing page template designed to increase conversion of your product. Backyard is flexible to suit any kind of your business. Try now and join with our happy customers!

Features
Subscribe

Available for:
iOS
Android

Toggle navigation

- About
- Features
- Subscribe
- Screenshots
- Reviews
- Download

## Optimize performance through advanced targeting solutions

Good marketing makes the company look smart. Great marketing makes the customer feel smart, - Joe Chernov. Never doubt a small group of thoughtful, committed people can change the world. Indeed, it is the only thing that ever has, - Margaret Mead. The best way to predict the future is to create it, - Peter Drucker.

Learn More

## Recreate your ideas and gain more success

Increase your user loyalty by maintaining mutual communication and nurturing your online community.

#### Marketing Strategy

Good marketing makes the company look smart. Great marketing makes the customer feel smart.

#### App Monetization

Content builds relationships. Relationships are built on trust. Trust drives revenue. - Andrew Davis

#### Store Optimization

Never doubt a small group of thoughtful, committed people can change the world. Indeed, it is the only thing that ever has.

#### User Management

Instead of using technology to automate processes, think about using technology to enhance human interaction.

## Learn how to make your app marketing efficient

Good marketing makes the company look smart. Great marketing makes the customer feel smart, - Joe Chernov. Never doubt a small group of thoughtful, committed people can change the world. Indeed, it is the only thing that ever has, - Margaret Mead. The best way to predict the future is to create it, - Peter Drucker.

Download Now

We'll research the market, identify the right target audience, analyze competitors and avoid users churn to increase retention. Enter your email address and we will do the rest.

SUBSCRIBE

## SCREENSHOTS

## TESTIMONIALS

"We are very happy and satisfied with Backyard service. Our account manager is efficient and very knowledgeable. It was able to create a vast fan base within very short period of time. We would highly recommend Backyard App to anyone."

Jennifer Lopez, Microsoft

"Everything looks great... Thanks for the quick revision turn around. We were lucky to find you guys and will definitely be using some of your other services in the near future."

Mike Portnoy, Dream Theater

"Overall, the two reports were very clear and helpful so thank you for the suggestion to do the focus group. We are currently working with our developer to implement some of these suggestions."

Jennifer Love Hewitt, Lead Vocal

## Would like to know more?

We'll research the market, identify the right target audience, analyze competitors and avoid users churn to increase retention. Download now for free and join with thousands happy clients.

- Available on theApp Store
- Available on thePlay Store


Copyright Backyard 2015 - Template by MoxDesign
